# A Practical Treatise on the Diseases of Children, from Birth to the Period of Puberty

**Published:** 1843-04

**Authors:** 


ART. III.

1. *Traité Pratique des Maladies des Enfants, depuis la Naissance jusqu'à la Puberté.* Par E. A. J. BERTON, D.M. &c. 2ième édition, entièremente refondue.—*Paris*, 1842. 8vo, pp. 823.

*A Practical Treatise on the Diseases of Children, from birth to the period of puberty.* By Dr. E. A. J. BERTON. Second edition, entirely rewritten.—*Paris*, 1842.

2. *Traité Pratique des Maladies de l'Enfance.* Par F. BARRIER, D.M. Tome Premier.—*Paris*, 1842. 8vo, pp. 735.

*A Practical Treatise on the Diseases of Childhood.* By Dr. BARRIER. Vol. I.—*Paris*, 1842.

THE diseases of childhood have of late years attracted peculiar attention in France. The immense hospitals of Paris afford an ample field for their investigation, and the large number of recent publications on the nature and treatment of these affections, bears testimony to the zeal with which it is cultivated. M. Valleix's *Clinique des Maladies des Enfans*, which appeared in 1838, was followed in the succeeding year by the *Traité Pratique des Maladies des Enfans* of M. Richard of Nancy. In the year 1841, a monthly journal for children's diseases was set on foot,* and 1842 had scarcely commenced when the books whose titles are at the head of this article made their appearance, both thick and closely printed octavos, and one being only the first volume of a work which will probably extend to two more before its completion. M. Becquerel, the laborious *ex-interne* of the Hôpital des Enfans Malades, has published the first number of a treatise on the diseases of children, which is to appear in parts, and a complete work on the subject by MM. Rilliet and Barthez, so favorably known by their essay on pneumonia in children, is in the press, and will be published before the close of the year.

Valuable as many of the observations are which these works contain, we yet cannot suppress a feeling of disappointment at the smallness of the results which have followed so much labour. Instead of confining themselves to the patient investigation of some one disease, these writers have taken a wider range, and have treated of all the affections incidental to childhood. They have presented us not with monographs, but with so-called complete theoretical and practical treatises, which abound in facts incompletely observed, and in conclusions drawn hastily from insufficient premises, and which lead us to a region of doubt and uncertainty. The brief period of an *internât*, no small part of which must be spent in learning to observe, is far too short to qualify any one for writing on all the diseases of children, and the opportunities of after life are in most cases nadequate to supply this deficiency. Thus we find M. Berton publishng in 1842 a new edition of his Traité des Maladies des Enfans, which

* La Clinique des Hôpitaux des Enfans. 1re année.—Paris, 1841. 8vo.

contains three hundred pages more than the former one; but this addition consists of the opinions, worthless as well as valuable, of all who have written on the subject within the last seven years, not of the results of the author's greater experience and maturer judgment.

Both of the works now before us illustrate the defects of which we have been complaining; from both, however, much may be gleaned that is worth knowing, and to this more grateful task we now apply ourselves. In an introduction of fifty pages, M. Barrier propounds a theory of life, which he wishes should serve as our guide in the management of childhood and its diseases. The vital force, says he, is strongest in the fœtus, stronger in the infant than in the child, in the child than in the grown man. With the advance of age the instrument becomes indeed more perfect, but the power which formed it progressively decreases till death. Strangely enough, M. Barrier seems to fancy this theory to be a novelty; a discussion of its merits would be out of place here, but the fact of the vital force being so strong at the age of virility as to multiply itself by the formation of germs, affords a most powerful argument against a hypothesis of a gradual and progressive waning of the vital force from the period of birth.

Turning from speculation to fact, we light upon some remarks by M. Barrier on the comparative prevalence of different diseases in childhood. His observations are too few to warrant any positive conclusions, but they will be interesting for comparison with more numerous data at some future time. It appears that of 412 cases which occurred in male children between April and October, 1838, two fifths were affections of the chest, one fifth of the abdominal viscera, one fifth of the organs of the senses; in one tenth the nervous centres were affected, and in one tenth various diseases existed not referrible to any of the above classes. Of diseases of the chest, pneumonia was by far the most frequent, and in three cases out of four it assumed the lobular form. Diarrhœa existed in half of the instances in which there was disorder of the abdominal viscera. The commonly received opinion of the great frequency of cerebral disease in childhood needs some qualification. Cerebral affections are frequent in childhood as compared with after years, but not as compared with the other diseases incidental to this period of life.

Early infancy is well known to be the time at which the mortality of the human race is highest; but it further appears that between the ages of two and fifteen, the greatest number of cases of illness occurs in the first and last years of childhood. Another fact which results from M. Barrier's researches is, that among a given number of sick children the tendency of their diseases to complications is in a direct ratio to the youth of the patients. The age of children likewise influences their liability to different diseases, as is shown by the following table, which represents the frequency of different classes of affections at three periods of childhood.

| | Years. From 2 to 5 | Years. 5 to 8 | Years. 8 to 15 |
|---|---|---|---|
| 1. Diseases of the chest | 42 | 46·50 | 40 |
| 2. ,, abdomen | 22 | 12 | 25 |
| 3. ,, nervous centres | 9 | 6·50 | 5 |
| 4. ,, organs of the senses | 21 | 26 | 19 |
| 5. ,, various parts | 6 | 9 | 11 |
| | 100 | 100 | 100 |

In further elucidation of this table, M. Barrier goes into particulars at p. 14, which we pass over. His fourth class is very unfortunately selected, including as he does under it all the eruptive fevers, as well as diseases which could strictly be called those of the organs of the senses.

We have on former occasions alluded to the high mortality at the Hôpital des Enfans Malades, and to the tendency of diseases occurring there to assume a complicated form. M. Barrier confirms the statements of previous writers, and likewise enters into an inquiry as to the causes of this mortality, some of which appear to admit of an easy removal, while others are, we fear, inseparable from an hospital for children. The cold in winter, and the heat of summer, together with the stinging of the gnats which infest the wards and keep the children in a state of constant irritation, operate very unfavorably upon the patients. The impossibility of varying the diet so as to suit the caprices of the children seems not to be without injurious effects, while very mischievous results follow from the ill-judged fondness of relations, who bring sweetmeats and pastry into the hospital on visiting days. The absence of a distinct ward for the eruptive fevers has had a very unfavorable influence on the mortality in the institution, those diseases frequently attacking patients admitted for other affections, in which case they invariably prove more fatal than when they constitute the primary affection. The want of a sufficient number of nurses, however, is the greatest defect at the Hôpital des Enfans. In a ward containing forty-five beds, the attendants consist of one sister and three nurses, and at night the whole charge of the patients is entrusted to one female. It is easy to conceive the evils which must result from such a system, and the great good which would be produced by increasing the number of nurses, but we doubt whether it is possible without an enormous expenditure of money to afford to young children suffering under acute diseases as good a chance of recovery in the wards of an hospital as they would have in the homes of their parents.

Two hundred pages of M. Barrier's book are occupied with the consideration of pneumonia, but we find in it little which needs mention after our recent notice of the treatise of MM. Rilliet and Barthez. There is, however, one point of great practical importance to which both M. Barrier and M. Berton refer, namely, the frequency of pneumonia unaccompanied by any well-marked symptoms, or marked by those which are characteristic of diseases of other organs than the lungs.

"In many cases," says M. Berton, "this disease appears under the form of a simple acute bronchitis, and runs its course in as short a time as that malady: in other instances the degree of emaciation to which it reduces the patient, and the slowness of its advance towards a fatal termination, might lead any one to mistake it for phthisis. This is the case more especially when pneumonia attacks patients of weak constitution and in feeble health, when it coexists with other affections and advances in a latent manner; circumstances by no means unusual, since it seldom happens that marks of inflammation of the lung are found in young subjects, without other lesions being discovered elsewhere. Numerous affections either usher in or come on during the course of pneumonia in children: The complication of inflammation of the lungs with cerebral symptoms is by no means unusual, and always of very serious nature." (Berton, p. 373.)

The complication of pneumonia with gastro-enteritis we believe to be much less common in this country than at the Hôpital des Enfans. Its coexistence with cerebral symptoms, so grave as to throw the pulmonary affection quite into the background, is, however, by no means rare. The difficulty of diagnosis in this case depends, as M. Berton correctly remarks, much on the class of symptoms which made their appearance first. If the pneumonia be consecutive it often becomes developed, and makes great advances unnoticed, while the attention of the practitioner is engrossed by the symptoms indicative of disturbance of the nervous centres. In these cases the inflammation of the lung betrays itself only by a slight cough, and even that sign is sometimes wanting. The medical attendant directs his treatment to the removal of the cerebral affection, and while congratulating himself on having been successful, has the mortification to lose his patient by a disease the existence of which he had not suspected, or which at any rate he had supposed to be nothing more than a trivial complication of the graver malady.

Delirium and coma are almost the only forms of cerebral disturbance which coexist with pneumonia in the adult. In the child, however, the cerebral symptoms are much more varied. Violent convulsions sometimes occur, accompanied with twitchings of the limbs or paralysis of some of the extremities, and occasionally those symptoms come on without previous convulsions. Sometimes the symptoms so closely resemble those of ordinary hydrocephalus, that, without having recourse to auscultation, the most acute and well-practised observer might be deceived, and yet on examining the body after death, the brain appears quite natural, or a slight and scarcely appreciable congestion is the only point in which it differs from a perfectly healthy state. But, difficult as the diagnosis of pneumonia may be in some cases, that of pleurisy is often still more obscured by similar causes. The head affection which accompanies it, and often ushers it in is more violent than in pneumonia; the child's restlessness renders auscultation less easy, and the signs which auscultation furnishes are less clear and more readily overlooked than those which announce inflammation of the lung.

Pleurisy is not, as the statement of some recent writers might lead the student to suppose, a disease of rare occurrence in the young subject. The laborious dissertation of M. Baron, some of the results of which are presented to us by M. Berton (p. 447), has set at rest the question on which very conflicting opinions had prevailed. He met with traces of pleurisy in the bodies of 159 out of 403 children, or in rather more than one in three. From his researches it further results that the frequency of pleurisy differs greatly at different periods of childhood; that it occurs oftener during the first five days after birth than at any other time in the first month of infancy; that it diminishes in frequency from the first month to the second year; that from the second to the third year it is more frequent than from the third to the fourth, or from the fourth to the fifth year. It continues rare during the remainder of childhood, and is especially so from the thirteenth to the fourteenth year. M. Barrier (p. 243), whose observations were made among children from two to fifteen years of age, states that he has never met with a case of pleurisy unconnected with pneumonia in children under six years old; that from six to ten it is rare; but that from ten to fifteen it is as frequent as in the adult. The number of cases from which he draws this conclusion is not stated by M. Barrier.

M. Berton's work is decidedly of far greater value than that of M.

Barrier, though one of the most unreadable books we have ever met with. It is impossible to present our readers with an analysis of it, since it contains above 120 cases of different diseases, some well, others ill reported, and which all seem to have been transferred from the writer's note-book without selection or revision. A passion for publishing cases, similar to that which has taken possession of the French school, prevailed throughout Europe from the middle of the fifteenth to the middle of the sixteenth century. The visitors to our public libraries will notice large folio volumes, *centuries of observations*, the result of these unwearied labours. If unacquainted with the history of medicine he would probably exclaim, "Surely the writers of these books are the men to whom science is indebted;" but he would soon learn that their works did not advance knowledge, and that the writers have fallen into oblivion, while Sydenham, who wrote comparatively little, who has not detailed a single case at length, did more than them all for the furtherance of our art, and has earned to himself a name which will never be forgotten. We want the fruits of observation; mere cases are valuable only as proofs that the writer has not formed his opinions on insufficient grounds, or as illustrating some point of peculiar importance, or, lastly, as furnishing pictures of disease more graphic and more faithful than a person could draw from memory. M. Berton has given us cases in abundance; he has also given more than enough of the opinions of others; his own we are not unfrequently left to pick out here and there as we best can.

Cerebral affections are the first class of diseases which he notices. The opinions of different writers on the anatomical characters of hydrocephalus are passed in review by him. He refers to the frequent existence in this disease of softening of the central parts of the brain, and recognizes the inflammatory character of that variety which is marked by a rose tint of the cerebral substance. He hesitates as to the class to which the *ramolissement blanc* should be referred. The valuable researches of M. Durand Fardel, which go far towards establishing its intimate connexion with the *ramolissement rouge* and its inflammatory origin, were, if we mistake not, unpublished at the time of the appearance of his treatise.

Divisions of hydrocephalus into various stages, distinguished by peculiar conditions of the pulse or by other signs have been proposed from time to time, and some, as that of Whytt, have been extensively adopted. M. Berton points out, at p. 128, the little regularity in the alterations of the pulse on which Whytt founded his classification, while not unfrequently the threefold modification in the beat of the arteries does not occur at all. There is usually, in cases of hydrocephalus, a premonitory stage in which the symptoms of irritation are present, many of them indeed are common to other diseases, but some, as the headach, the regurgitation of food, and the vomiting without effort, are characteristic of this affection. A second period, in which these signs are more fully developed, succeeds, and is followed by one marked by the phenomena which indicate the near approach of death in all acute diseases, to which are superadded others dependent on the organic lesions of the brain. It is of importance, however, to bear in mind that hydrocephalus often runs its course without any full exhibition of its peculiar symptoms. No other signs appear than those which showed themselves at the beginning of the attack, till as life begins to fail, various signals of constitutional distress announce the impending dissolution. At other times no first stage is evident, the malady runs its course so rapidly, that it seems to have begun with the second stage. But, besides this irregularity in the course of the disease, its symptoms are occasionally so little marked, that its nature might easily be altogether mistaken.

"We have sometimes met with no other symptom, even in children from four to five years old, than fever, vomiting, a sensation of heat about the supra-orbitar region appreciable to the hand; low spirits, pettishness, and a kind of taciturnity, with somnolence, or constant desire to sleep. In two cases of this kind which came under our notice last year, the continual vomiting might have led to the suspicion of some disease of the stomach, but its existence was disproved by a post-mortem examination." (Berton, p. 129.)

Some valuable observations of this kind were related by M. Trousseau, in his clinical lectures, from which M. Berton quotes the following;

"A child was admitted into the hospital, which seemed rather disinclined to move, but continued to suck, and had no convulsions until the day before it died, when its head was thrown somewhat back. On a post-mortem examination we found a great number of tubercles in the pia mater, and softening of the superficial layer of the brain. This child died of tubercular meningitis, but presented no sign of convulsions except slight throwing back of the head.

"A little girl seven years old, who lived at Paris, told her mother one day that she could see only half of a child who was at play a short distance from her: she had been struck with hemiplegia. Some days afterwards she complained of being disturbed by the noise of the carriages in the street, and of the children at play; she next showed her fondness for her parents in a manner which was not customary with her. Vomiting, fever, and slight drowsiness followed. A week afterwards the child fell into a sleep so deep that it was impossible to wake her; the conjunctiva became injected, convulsions occurred, and she died. Towards the close of the illness, the cerebral disease was very manifest, but at its commencement nothing could have excited suspicion of the nature of the affection.

"Another little girl, five years old, who lived with her parents in a house in the country, eleven leagues from Paris, complained of cold (it was in the month of June) and of weariness; she returned home, went to bed, and fell asleep. In the evening she was seized with a shivering fit, which recurred about the same hour on several successive days. The medical practitioner of the place was sent for; he took the case for one of intermittent fever, and gave the sulphate of quinine, but without benefit. At the end of a week there was marked exhaustion, and the child made almost constant efforts to vomit. The mother now became uneasy about her daughter, and having brought her to Paris we were sent for. We found the little patient in such a state of stupor that she did not notice us. M. Récamier was at once called to meet us in consultation, and, on account of the loaded condition of the tongue, he ordered an emetic of ipecacuanha. On the following day the index finger of one hand was paralysed, on the day after there was paralysis of one eyelid. Death took place after the lapse of some days, and, on examining the body, we found softening of the surface of the brain, and a little turbid serum in the ventricles." (Berton, pp. 129-32.)

In our earlier practice we treated a fatal case of hydrocephalus, the exact nature of which was so marked by incessant and extreme vomiting, that we could not be persuaded of its real character until we saw the ventricles full of fluid after death.

Cerebral congestion is one of the most common diseases of infancy, but cerebral hemorrhage is an occurrence of extreme rarity. M. Berton states (p. 217,) that during twenty years, M. Guersent has met with but two instances of it, either in private practice or at the Hôpital des Enfans, and there are only nine observations of it on record. Insensibility, stertor, and paralysis of one side coming on suddenly, are its characteristic symptoms, while contractions of the limbs, and convulsive movements, are the usual indications of cerebral congestion. Distinct from hemorrhage into the substance of the brain, is hemorrhage into the cavity of the arachnoid, an occurrence by no means rare in the new-born infant, of which representations are given in M. Cruveilhier's Anatomie Pathologique. M. Serres has likewise described what he calls *apoplexie méningée*, which would appear to be a form of acute meningitis, terminating in hemorrhage on the surface of the brain; and this, too, is not an affection of great rarity. Since the publication of M. Berton's work, a paper on cerebral hemorrhage has appeared from the pen of M. Becquerel, (Clinique des Maladies des Enfans, 15 Avril, 1842,) in which a distinction is drawn between simple hemorrhage into the substance of the brain, and that which occurs as a complication of tubercular disease of the brain or pia mater. This latter form of hemorrhage is by no means so rare as the other; it usually takes place in the midst of the softened cerebral substance which is often found around tubercular deposits.

Selecting, as we proposed to do, such subjects only as seemed to present the greatest interest, we pass over several chapters, till we come to croup, of which both M. Barrier and M. Berton have treated. Both these gentlemen, but especially the former, appear not to have distinguished sufficiently between croup, such as is met with in this country, and diphthérite; M. Barrier, indeed, seems to regard the two diseases as identical. The diphthérite of the French writers is in all probability identical with the *garotillo* of the Spaniards, which formerly prevailed epidemically at various times, especially in the southern parts of Europe. The sore throat, the difficulty of swallowing, the great depression of strength, and the assumption of an epidemic character, are common to the two diseases. We find, however, in the older medical writers, notices of an affection of the air-passages quite different from the *garotillo* or *diphthérite*. In 1517 a catarrhal affection prevailed throughout Germany and the Netherlands, the symptoms of which closely resembled those of croup, while at the same time an epidemic raged at Basle, in which the tongue and fauces were white, as though coated with mildew; the patients were unable to eat or drink, and the local malady was associated with a pestilential fever, which proved extremely fatal. Again, in describing an affection of the throat, which prevailed epidemically at Alkmaar in 1557, Florestus (Lib. vi. Observ. i. & ii.) expressly remarks, "At noster hic epidemius morbus, nihil commune cum angina habebat, licet in gutture per modum catarrhi populariter grassaretur." We have little doubt but that careful research would find among the older writers mention of two distinct affections, in one of which the pharynx was involved, while in the other the disease was exclusively confined to the air-passages. Another distinction between true croup and diphthérite is found in the circumstance, that though both may prevail epidemically, yet that epidemics of the former disease usually affect but a small number of persons, while epidemics of diphthérite are generally very extensive.*

* In proof of this, see Eble, Versuch einer pragmatischen Geschichte der Arzneikunde, Band i. § 321—Wien, 1840; and M. Boudet's paper on Croup in the Archives Générales de Médecine for April, 1842.

The propriety or impropriety of performing tracheotomy in croup, is still a much-disputed question; but the extensive trials which have been made of it by M. Trousseau, appear to be winning favour for it in France. M. Barrier states (p. 440,) that at the end of May, 1840, M. Trousseau had performed tracheotomy in 102 children affected with croup, of whom he saved 26, or one in four. This result is favorable to tracheotomy, since M. Guersent estimates the number of recoveries in cases treated without the operation at one in five. With reference to the time at which tracheotomy should be performed, M. Trousseau is decidedly in favour of its being resorted to at an early period, and of all his cases those did best in which no treatment whatever had preceded the operation.

M. Berton quotes from M. Gendron's thesis on Bronchotomy the following observations on the chances of success or failure of the operation under different circumstances:

"We are of opinion," says M. Gendron, "that practitioners frequently hesitate to perform this operation, owing to the slender chances of recovery which it often presents; some of the circumstances which would warrant a person in practising it without hesitation may be worth mention. Whenever the voice becomes stifled, the respiration hissing, when the patients make great muscular efforts in expiration, when the cough is seldom and suffocation imminent, and the face has lost its natural colour, it is probable that the approaching asphyxia results from some obstacle to the passage of the air in the larynx itself; and in such cases, opening the trachea reestablishes respiration, and permits the application of caustic to those surfaces which the diphthérite has reached. The operation still presents some chances of success when the cough, more frequent but loose, announces bronchial croup; the looseness of the cough gives reason to hope that the flakes of false membrane will be speedily detached, and a twofold way is thus opened for their escape. If, on the contrary, the voice and respiration are dry and insonorous, if the voice had assumed the stifled character long before the first paroxysm of suffocation, if the inspiration has a blowing rather than a hissing sound, and the expiration is short, quick, and easy; if, in spite of the hoarseness, the patient's articulation is distinct, and effected without any great effort of the muscles of the neck; if the chest is largely dilated, the asphyxia is bronchial, the false membrane is adherent, tracheotomy would produce no change whatever in the respiration, and would not even have the advantage of retarding the patient's death." (Berton, p. 320.)

There is another disease of the air-passages,—coryza,—not without danger to infants at the breast, though usually of no moment to such as are a few years old. The principal danger arises from the formation of pseudo-membranous excretions, which were found by Billard, in five out of forty cases, lining the nares, and adhering very firmly to the membrane, which was of a bright red colour, thickened and friable. In mild cases of the disease hardly any treatment is required, but in those where the head seems affected, a blister may be applied to the nape of the neck, or leeches may be employed if there should be any well-marked cerebral congestion. If the disease is attended by the formation of a false membrane, insufflation of equal parts of powdered alum and sugar-candy is recommended by M. Berton (p. 356), or if it assumes a more chronic form, injections of various kinds may be thrown up the nostrils, or the surface of the nares may be touched with a solution of nitrate of silver.

In M. Berton's work is embodied a dissertation on tubercle of the bronchial glands, which gained for its author the medal of the *Société Médicale d'Emulation*, in 1830. The reader will find much in it that is of interest, but the field of inquiry to which it leads is too vast for us to enter on at the present time, besides which the essay is reprinted without alteration, as it appeared in the first edition of the work, and therefore does not contain anything absolutely new. The subject of tubercle and scrofula occupies the last 200 pages of M. Barrier's book. His remarks are characterized by sound sense, and the supposition which he advocates of there being certain scrofulous changes of organs quite independent of the presence of tuberculous matter, is one which many circumstances favour. We know no place where the whole subject of scrofula and tubercle could be better studied than at the Hôpital des Enfans, and for one good essay upon it, containing the results of careful investigation, and not written to develop one idea, or to serve some favorite theory, we would gladly barter, and we say it without intending to disparage the merits of M. Berton or M. Barrier, whole volumes of Practical Treatises on children's diseases, such as issue from the Paris press.

---

